# Multicomponent Nanoparticles Synergistic One-Dimensional Nanofibers as Heterostructure Absorbers for Tunable and Efficient Microwave Absorption

**DOI:** 10.1007/s40820-022-00986-3

**Published:** 2022-12-15

**Authors:** Chenxi Wang, Yue Liu, Zirui Jia, Wanru Zhao, Guanglei Wu

**Affiliations:** 1 Institute of Materials for Energy and Environment, State Key Laboratory of Bio-Fibers and Eco-Textiles, College of Materials Science and Engineering, Qingdao University, Qingdao, 266071 People’s Republic of China; 2 College of Chemistry and Chemical Engineering, Qingdao University, Qingdao, 266071 Shandong People’s Republic of China; 3 Weihai Innovation Institute, Qingdao University, Qingdao, 264200 Shandong People’s Republic of China

**Keywords:** Electrostatic spinning, Necklace-structured nanofibers, Broadband response, Electromagnetic wave absorption

## Abstract

Application of novel radio technologies and equipment inevitably leads to electromagnetic pollution. One-dimensional polymer-based composite membrane structures have been shown to be an effective strategy to obtain high-performance microwave absorbers. Herein, we reported a one-dimensional *N*-doped carbon nanofibers material which encapsulated the hollow Co_3_SnC_0.7_ nanocubes in the fiber lumen by electrospinning. Space charge stacking formed between nanoparticles can be channeled by longitudinal fibrous structures. The dielectric constant of the fibers is highly related to the carbonization temperature, and the great impedance matching can be achieved by synergetic effect between Co_3_SnC_0.7_ and carbon network. At 800 °C, the necklace-like Co_3_SnC_0.7_/CNF with 5% low load achieves an excellent *RL* value of − 51.2 dB at 2.3 mm and the effective absorption bandwidth of 7.44 GHz with matching thickness of 2.5 mm. The multiple electromagnetic wave (EMW) reflections and interfacial polarization between the fibers and the fibers internal contribute a major effect to attenuating the EMW. These strategies for regulating electromagnetic performance can be expanded to other electromagnetic functional materials which facilitate the development of emerging absorbers.

**Supplementary Information:**

The online version contains supplementary material available at 10.1007/s40820-022-00986-3.

## Highlights

- Heterogeneous interface engineering is designed by electrospinning.
- The introduction of Co_3_SnC_0.7_ nanoparticles increased the loss mechanism.
- Enhanced electromagnetic loss and improved impedance matching are achieved.
- The absorbers exhibit high-efficient electromagnetic wave absorption performance.

**Supplementary Information:**

The online version contains supplementary material available at 10.1007/s40820-022-00986-3.

## Graphical abstract

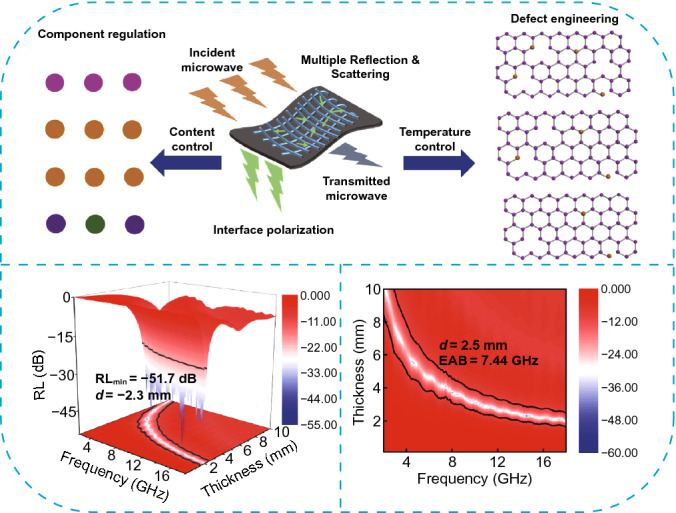

## Introduction

During the past decades, the large amounts of electronic devices have enriched people's life. However, it also increases the risk of our body exposure to the electromagnetic wave (EMW) [1–5]. Excessive electromagnetic radiation has not only become another major source of environmental pollution, but also lead to profound health problems of biont. Therefore, searching for a material that can attenuate EMW radiation and reduce the harm of EMW has become a focal point in materials science research [6–10]. Related studies have shown that the EMW properties of carbon-based composites are related to their nanostructures, and rational design of carbon-based composites is expected to achieve excellent wave-absorbing properties [11, 12]. There were many reports that materials with hierarchical construction such as yolk–shell [13], porous [14], and hollow nanostructures [15], display a significantly enhanced and even frequency-tunable EMW absorption performance. Although the carbon nanoparticles occupy scores of advantages, structural constraints such as zero-dimensional cannot form as efficient conducting networks like one-dimensional structures [16–19].

Polymer-based fiber composite films have attracted much attention in batteries, supercapacitors, photocatalytic degradation, and wearable sensors, because of the special properties of carbon-based composites, and the electrical and thermal conductivity of metal materials, the heat resistance, and corrosion resistance of ceramic [20–23]. Therefore, traditional melt-spinning, solution-spinning, and gel-state spinning processes always produce the fibers in the micrometer scale, while nanoscale fibers are continuously extruded through the high electrostatic fields of electrospinning. When the electric field force reaches a certain strength, the spinning droplet will overcome its own surface tension to form a charged jet. After the solvent evaporates or solidifies, the fibers are collected on the receiving device to shape a fiber mat. Nano-scale fibers prepared by electrospinning usually show a larger specific surface area, a wider porosity, and excellent flexibility. As an electromagnetic wave-absorbing material, single-component carbon nanofiber has the disadvantages of low permeability, poor absorption strength, and narrow absorption bandwidth [24]. When carbon fiber is composited with alloys or metal oxides, it will strengthen the EMW absorption performance caused by the single loss mechanism. Zhang et al. [25] reported that the NiCo_2_O_4_ nanofiber integrates electromagnetic absorption and electromagnetic shielding. The electrical conductivity can be tuned by controlling the internal structure of the fibers to achieve electromagnetic wave absorption or shielding or both under specific conditions. Yang and his colleagues [26] prepared hierarchical composite fibers (HCF@CZ-CNTs), which achieved an effective bandwidth of − 53.5 dB at 2.9 mm. Wu et al. [27] used PVP to convert the precursor ZIF-67 into cobalt-layered double-hydroxide/carbon fiber with a maximum effective bandwidth of 6.5 GHz at 2.0 mm. These multi-component one-dimensional structures can improve the electromagnetic wave absorption performance by adjusting the component ratio in multiple dimensions. One-dimensional materials benefiting from anisotropy and high aspect ratio will easily form carrier transport paths in the axial direction under electromagnetic fields [28–30].

In this work, we reported the fabrication process of Co_3_SnC_0.7_ nanoparticles encapsulated in carbon nanofibers as absorbers. The metal oxide CoSnO_3_ is prepared by wet chemical method and dispersed in the spinning solution for electrospinning to achieve the purpose of uniform distribution of nanoparticles. The calcination temperature determines the thermal movement of metal ions and the degree of carbonization on the fiber surface during the thermodynamically controlled crystallization process. And the change of the dielectric and magnetic properties about CoSnO_3_/PANF with different content of CoSnO_3_ after calcination at 800 °C was investigated. The rationally designed necklace-like Co_3_SnC_0.7_/CNF may provide a new strategy to extended to other lightweight electromagnetic wave absorbers.

## Experimental Procedure

### Materials

Cobalt (II) chloride hexahydrate (CoCl_2_·6H_2_O, AR), tin tetrachloride pentahydrate (SnCl_4_·5H_2_O, AR), trisodium citrate dihydrate (C_6_H_5_Na_3_O_7_·2H_2_O, AR), sodium hydroxide (NaOH, AR), absolute ethanol (CH_3_CH_2_OH, AR), were purchased from Sinopharm Group Chemical Reagent Co., Ltd. *N*, *N*-Dimethylformamide (C_3_H_7_NO, AR) was purchased from Shanghai Aladdin Biochemical Technology Co., Ltd. Polyacrylonitrile ((C_3_H_3_N)_n_, average Mw = 85,000) was provided by Shanghai Macklin Biochemical Co., Ltd. The above samples can be used without purification.

### Preparation of CoSnO_3_/PANF

The entire preparation method of CoSnO_3_ was reported in our previous work, CoSn(OH)_6_ was prepared by a wet chemical method, and CoSnO_3_ particles were finally formed after annealing [31]. CoSnO_3_/PANF was used as precursor by electrospinning technology. Two hundred milligram CoSnO_3_ nanocubes were dispersed in 10 mL of N, N-dimethylformamide (DMF) solution and stirred for 1 h under sonication to form a suspension. In order to obtain the spinning solution, 1 g of polyacrylonitrile was added to the suspension and the mixture stirred at 60 °C for 5 h and followed by transferring into a syringe linked with a 19-gauge pinhead. With a speed of 0.3 mL h^−1^ at the ultrahigh voltage of 18 kV, CoSnO_3_/PANF was collected by release papers, which were situated 10 cm from the pinhead.

### Synthesis of the Co_3_SnC_0.7_/CNF-700, Co_3_SnC_0.7_/CNF-800, Co_3_SnC_0.7_/CNF-900, and CNF

The obtained CoSnO_3_/PANF was heated in muffle furnace at 270 °C for 2 h and transferred into a tubular furnace. The Co_3_SnC_0.7_/CNF-700, Co_3_SnC_0.7_/CNF-800, and Co_3_SnC_0.7_/CNF-900 were synthesized at 700, 800, and 900 °C, respectively, under Ar atmosphere with a heating rate of 5 °C min^−1^. The CNF material was formed by carbonization treatment of PANF at 800 °C, and the other details were similar to Co_3_SnC_0.7_/CNF-800 (the samples of CoSnO_3_/PANF with 100, 300, 400, and 500 mg CoSnO_3_ doping amount after calcination at 800 °C were defined as S100, S300, S400, S500, respectively).

### Materials Characterization

The microstructure and morphology of as-preparation samples were characterized by field emission scanning electron microscopy (F-SEM; JEOL JSM-7800F) with equipment of element mapping. The lattice structure of Co_3_SnC_0.7_/CNF-800 composites was characterized by transmission electron microscopy (TEM; JEOL JEM-2100). The crystalline structure of sample was described by the powder X-ray diffraction (XRD, Rigaku Ultima IV with Cu-Ka radiation, *λ* = 0.15418 nm) in the range of 5°–90°. Use a Renishaw inVia Plus Micro-Raman spectroscopy system with a 10-Mw DPSS laser at 532 nm to test the Raman spectra in 300–1800 cm^−1^. X-ray photoelectron spectroscopy (PHI5000 Versaprobe III XPS) used to analyze the element composition, valence, and chemical environment of the sample. Thermal gravimetric analyzer (TGA) used to measure the relationship between sample mass and temperature. The samples of CoSnO_3_/PANF, CNF, Co_3_SnC_0.7_/CNF-700, Co_3_SnC_0.7_/CNF-800, and Co_3_SnC_0.7_/CNF-900 were uniformly dispersed in a paraffin matrix with the same loading ratio of 5 wt%, and the ring samples (*φ*_out_: 7.00 mm, *φ*_in_: 3.00 mm) were studied by a network analyzer (E5063A) at a frequency of 2–18 GHz, and the height is about 2 mm. The EMW reflection loss of materials enables to be calculated based on the measured data of EM parameters as follows [32–34]:

1 $$RL\left( {{\text{dB}}} \right) = 20\log \left| {\frac{{Z_{{{\text{in}}}} - Z_{0} }}{{Z_{E} + Z_{0} }}} \right|$$

2 $$Z_{{{\text{in}}}} = Z_{0} \sqrt {\frac{{\mu_{r} }}{{\varepsilon_{r} }}} \tanh \left( {j\frac{2\pi fd}{c}\sqrt {\varepsilon_{r} \mu_{r} } } \right)$$

where *Z*_in_ represents absorbers’ input impedance, *f* is the frequency of the EMW, *c* is the propagation speed of light, *d* is the absorbers’ thickness, and *Z*_0_ is the impedance in free space.

## Results and Discussion

### Structure Characterization and Performance Analysis

**Fig. 1:**
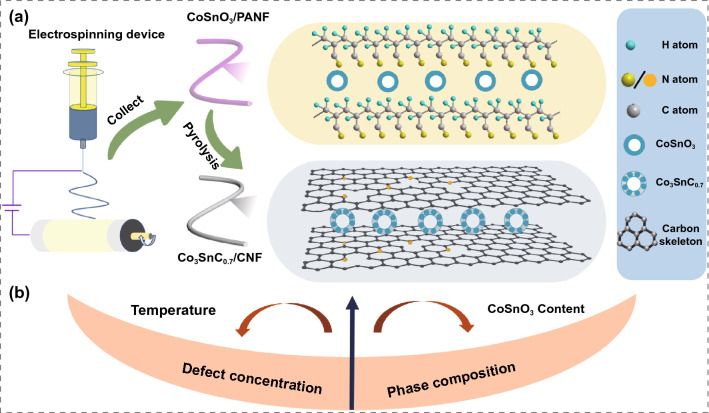
Schematic illustration of the synthesis of Co_3_SnC_0.7_/CNF (**a**); the relationship between temperature and content on defect concentration and composition of samples (**b**)

**Fig. 2:**
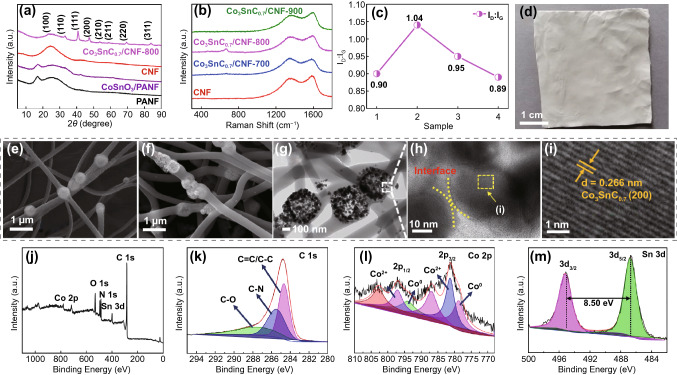
The XRD pattern of PANF, CoSnO_3_/PANF, CNF, and Co_3_SnC_0.7_/CNF-800 (**a**); Raman of CNF (Sample 1) and Co_3_SnC_0.7_/CNF-700 (Sample 2), Co_3_SnC_0.7_/CNF-800 (Sample 3), and Co_3_SnC_0.7_/CNF-900 (Sample 4) (**b, c**); the digital image of CoSnO_3_/PANF (**d**); the SEM images of CoSnO_3_/PANF (**e**), Co_3_SnC_0.7_/CNF-800 (**f**); TEM: low (**g**) and high definition (**h**) of Co_3_SnC_0.7_/CNF-800; XPS spectrum of sample Co_3_SnC_0.7_/CNF-800; full spectrum (**j**); fine spectrum: C 1*s* (**k**), Co 2*p* (**l**), Sn 3*d* (**m**)

As shown in Fig. 1a, the schematic illustration described a preparation process of the Co_3_SnC_0.7_/CNF composites. Temperature and CoSnO_3_ addition amount could control the defect concentration and component change of composites in different dimensions. The XRD pattern of PANF and CoSnO_3_/PANF is demonstrated in Fig. 2a, and there are similar diffraction peaks at approximately 17° which can be attributed to the (010) plane of PAN. The broad diffraction peak of CNF at 20°–30° represents the amorphous shape of carbon. Crystal planes distribution of Co_3_SnC_0.7_/CNF-800 is reflected in different peaks which located in 23.3°, 33.1°, 40.9, 47.5°, 53.8, 59.1, 69.7°, and 84.2°; these peaks can be indexed to the (100), (110), (111), (200), (210), (211), (220), and (311) crystal planes of Co_3_SnC_0.7_. Compared with JCPDS NO. 29-0513 card, all diffraction peaks shifted slightly to the left, which was caused by the uneven surface of the sample in the glass slide. The clearer XRD curve of Co_3_SnC_0.7_/CNF-700 and Co_3_SnC_0.7_/CNF-900 is indicated in Fig. S1, which corroborated with the crystal planes shown by the TEM image in Fig. S2. It indicates that the Co_3_SnC_0.7_/CNF composites are successfully prepared. The D-band and G-band within 1300–1600 cm^−1^ is relevant to the defect and graphitization of carbon. Two clear peaks at 1350 and 1580 cm^−1^ straightforwardly validate the presence of carbon fibers (Fig. 2b). Furthermore, the *I*_D_/*I*_G_ value of Co_3_SnC_0.7_/CNF-900 is 0.89, which reaches a lowest intensity ratio among all samples. This phenomenon suggests that high temperature can promote the transition of carbon fibers to higher graphite crystallinity, and the ratio of *I*_D_/*I*_G_ decreases with increasing temperature (Fig. 2c) [35, 36].

**Fig. 3:**
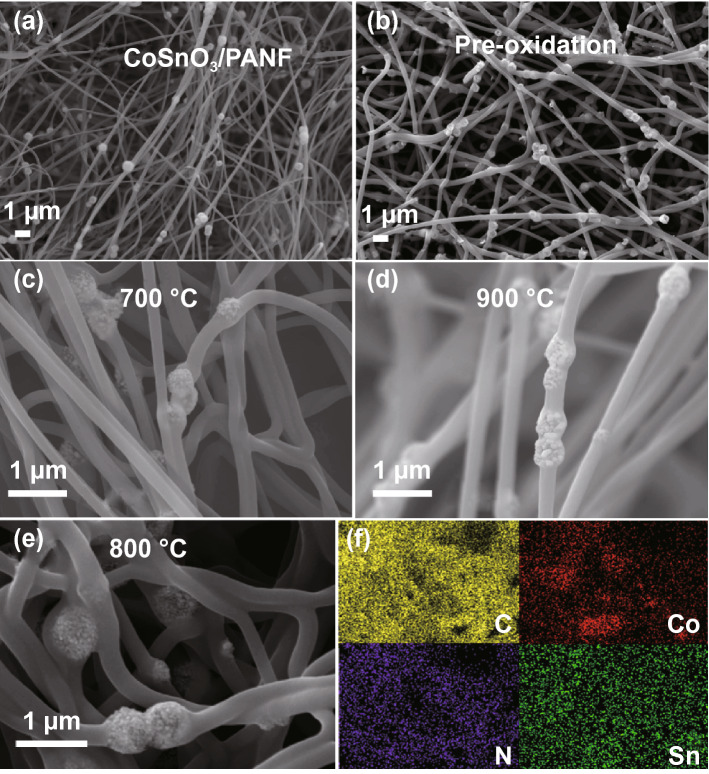
SEM images of CoSnO_3_/PANF (**a**), CoSnO_3_/PANF after pre-oxidation (**b**), Co_3_SnC_0.7_/CNF-700 (**c**), Co_3_SnC_0.7_/CNF-900 (**d**), Co_3_SnC_0.7_/CNF-800 (**e**); the element mapping of Co_3_SnC_0.7_/CNF-800 (**f**)

Necklace-like CoSnO_3_/PAN fibers were obtained by electrospinning of the prepared precursor solution, and the scattered CoSnO_3_ is evenly encapsulated in the PAN fibers, which is similar to the long necklace structure (Figs. 2e and 3a). The TG curve demonstrates the variation of the mass fraction of CoSnO_3_/PANF with the change of temperature (Fig. S3). When the temperature is lower than 280 °C, the quality of sample has hardly changed. This temperature is defined as the pre-oxidation temperature of the PAN fibers (Fig. 3b), and the mass loss of the sample mainly occurs in 300–500 °C. By pre-oxidation, the linear molecular chain structure of PAN fiber was changed into heat-resistant structure, which ensured the stability of the fiber under high temperature at argon atmosphere. After annealing of raw fiber at 800 °C, the low-magnification SEM image of Co_3_SnC_0.7_/CNF-800 is shown in Fig. 2f, and a large number of nanoparticles are generated on the surface of the cube template. The inner cubic shell of Co_3_SnC_0.7_/CNF-800 composite fiber still maintains the original morphology, and each element is evenly distributed in the composites (Fig. 3f). It can be seen from the SEM image (Fig. 3c) that the particle size of the sample Co_3_SnC_0.7_/CNF-700 is tiny and almost completely wrapped on the surface of the shell layer. The Co_3_SnC_0.7_ cubic structure is destroyed at the carbonization temperature of 900 °C (Fig. 3d). At the same time, defects in the carbon layer of the final product begin to increase as the increasement of temperature gradient. It originates from the thermal motion of atoms, resulting in the exfoliation of C atoms, the generation of vacancies, or replaced by N atoms. As a comparison, the microstructure of pure PAN fibers is listed in supporting information (Fig. S4). It is worth noting that the fibers surface transforms from wrinkled to smooth and porous structure after calcination, with a diameter of around 200 nm, which can be verified in Fig. S4b-d. TEM is utilized to further confirming the Co_3_SnC_0.7_/CNF-800 internal structure. Low-magnification TEM image (Fig. 2g) shows a hollow cubic box composed of small particles, which are attached to the inside of the fiber. In the high-magnification TEM images (Fig. 2h, i), the lattice fringe of Co_3_SnC_0.7_/CNF-800 is measured to be 0.266 nm, which matches with the (110) characteristic crystal plane of Co_3_SnC_0.7_.

XPS is one of the most effective methods to differentiate the varied of chemical band and states; the full-spectra of Co_3_SnC_0.7_/CNF-800 composites are shown in Fig. 2j. The C 1*s* spectrum (Fig. 2k) is composed of three peaks sited at 284.7 eV (*sp*^*3*^ C–C), 285.4 eV (C–N), and 287.2 eV (C–O). Noted that XPS full spectrum contains faint peaks of N and O, in response to the peaks of N 1*s* and O 1*s*. This can be attributed to the combined water in the air and the residual O and N after pyrolysis of the sample. In Fig. 2l, the two peaks associated with Co 2*p*_1/2_ and Co 2*p*_3/2_ are located at 781.3 and 797.0 eV with a binding energy difference of 15.7 eV. Co 2*p*_1/2_ and Co 2*p*_3/2_ can be deconvoluted into four distinct peaks after fitting, confirming the presence of Co^0^ and Co^2+^ species [37–39]. Figure 2m shows that the Sn 3*d* spectral exists two diffraction peaks located at 486.7 and 495.2 eV, which can correspond to Sn 3*d*_5/2_ and Sn 3*d*_3/2_. There is a binding energy difference of 8.50 eV between Sn 3*d*_3/2_ and 3*d*_5/2_; the result reveals the valence state of Sn (IV) [40].

**Fig.4:**
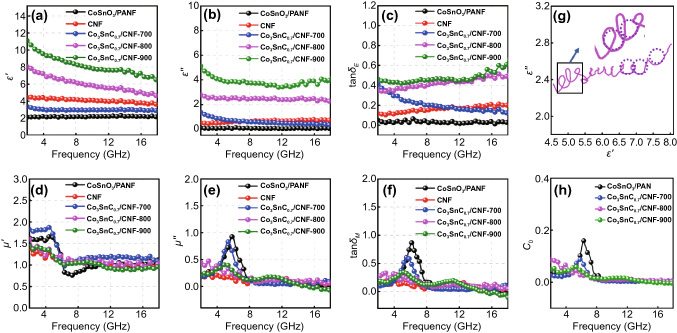
Real part (**a**) and imaginary part (**b**) of permittivity; the curves of tan*δ*_*E*_ (**c**); the real part (**d**) and imaginary part (**e**) permeability; the curves of tan*δ*_*M*_ (**f**); the Cole–Cole curve (**g**) of Co_3_SnC_0.7_/CNF-800 and the C_0_ curve (**h**) of CoSnO_3_/PAN, Co_3_SnC_0.7_/CNF-700, Co_3_SnC_0.7_/CNF-800, Co_3_SnC_0.7_/CNF-900

Complex permittivity (*ε*_*r*_ = *ε*′* − jε*″) and complex permeability (*μ*_*r*_ = *μ*′* − jμ*″) of absorbers can evaluate the ability of the attenuation to EMW. The real parts (*ε*′*, μ′*) are defined as the ability of energy storage for electromagnetic, and the dissipative electromagnetic energy ability is exhibited by imaginary parts (*ε*″*, μ*″) [41–44]. As shown in Fig. 4a, the poor conductivity of the CoSnO_3_/PANF results in the low complex permittivity, and it can be attributed to that the electrons on CoSnO_3_ surface cannot be transported freely across the fibers bundle. Therefore, the carbonized PAN fibers improve the real and imaginary parts of the dielectric, which can be attributed to the increase of electrical conductivity caused by the movement of charge carrier on the surface of the carbon shell. The dielectric parameter of all the Co_3_SnC_0.7_/CNF composites gradually increased with the increasement of the temperature. Co_3_SnC_0.7_/CNF-700 has the lower dielectric parameter, which is related to the poor degree of graphitization. The dielectric real part of Co_3_SnC_0.7_/CNF-800 and Co_3_SnC_0.7_/CNF-900 declined slowly at high frequency, which is related to the frequency dispersion effect in the samples. Magnetic loss is theoretically divided into three mechanisms (exchange resonance, natural resonance, and eddy current loss). It is clearly shown in Fig. 4e, f that the permeability parameters of CoSnO_3_/PANF, Co_3_SnC_0.7_/CNF-700, Co_3_SnC_0.7_/CNF-800, and Co_3_SnC_0.7_/CNF-900 fluctuate at low frequencies, which is related to the natural resonance occurring in the samples and can be exhibited by C_0_ curve in Fig. 4h [45–48]. The Cole–Cole curve can be used to indicate the polarization effect of absorbers, in which each semicircle represents a relaxation process. As shown in Figs. 4g and S5, the carbonized composites demonstrate significantly increased semicircles, especially in Co_3_SnC_0.7_–800, confirming the majority of polarization relaxation processes.

**Fig. 5:**
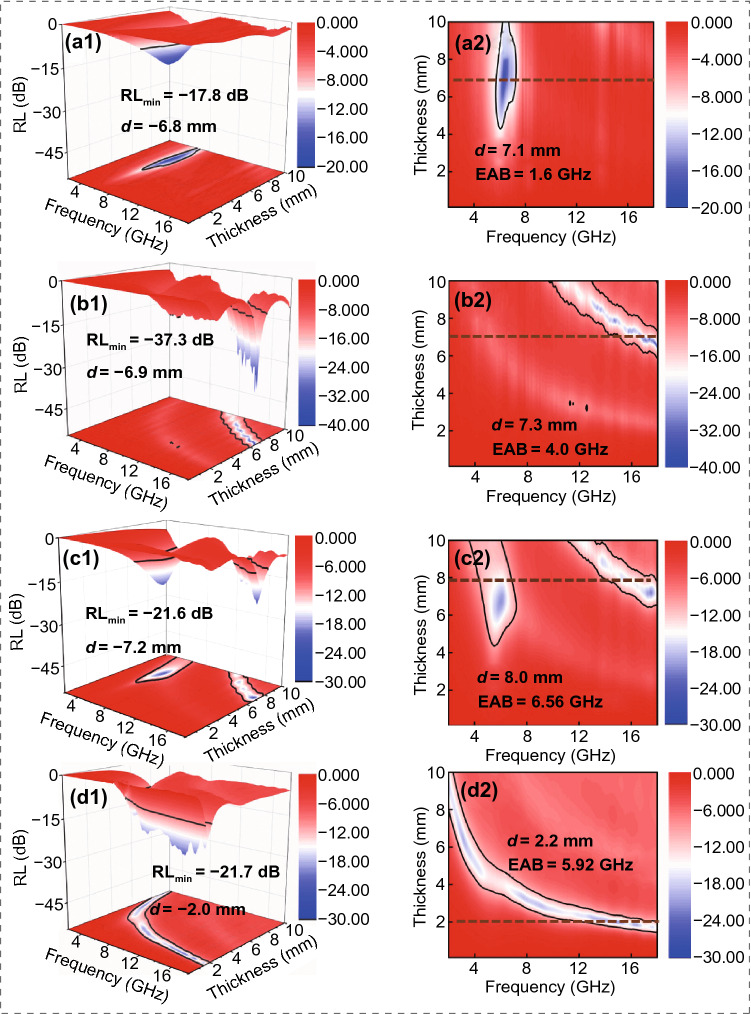
3D reflection loss of CoSnO_3_/PAN (**a1**), CNF (**b1**), Co_3_SnC_0.7_/CNF-700 (**c1**), Co_3_SnC_0.7_/CNF-900 (**d1**); the 2D effective bandwidth of CoSnO_3_/PAN (**a2**), CNF (**b2**), Co_3_SnC_0.7_/CNF-700 (**c2**), Co_3_SnC_0.7_/CNF-900 (**d2**)

**Fig. 6:**
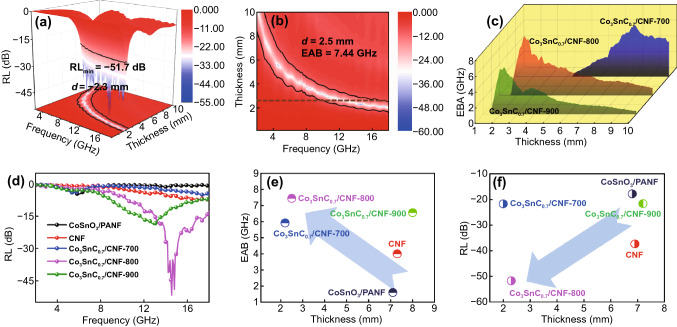
3D plots of reflection loss (**a**) and the bandwidth for Co_3_SnC_0.7_/CNF-800 (**b**); the 3D effective bandwidth of Co_3_SnC_0.7_/CNF-700, Co_3_SnC_0.7_/CNF-800, Co_3_SnC_0.7_/CNF-900 (**c**), and reflection loss of all samples at 2.3 mm (**d**); the relationship between effective bandwidth and matching thickness of five samples (**e**), the relationship between reflection loss and matching thickness of five samples (**f**)

From Fig. 5a1, a2, CoSnO_3_/PANF composites achieve the lowest *RL* of − 17.8 dB at 6.8 mm, and it is almost no effective bandwidth in 2–18 GHz. The poor electromagnetic attenuation ability can be attributed to the single loss mechanism of the precursor CoSnO_3_/PANF. In the same way, the sample of CNF achieves the lowest reflection loss (− 37.3 dB) at 6.9 mm (Fig. 5b1). It is shown in Fig. 5b2 that the effective bandwidth of CNF appears at high frequencies (12–18 GHz) with the thickness of 6–8 mm. It is worth noting that there is a thinner thickness (2.3 mm), and the maximum EAB of Co_3_SnC_0.7_/CNF-800 reaches 7.44 GHz from 10.40 to 17.84 GHz at 2.5 mm (Fig. 6a, b). As a comparison, the minimum *RL* of Co_3_SnC_0.7_/CNF-700 is − 21.6 dB at 7.2 mm and the maximum *EAB* is 6.56 GHz at 8.0 mm (Fig. 5c1, c2). Although the Co_3_SnC_0.7_/CNF-900 is improved in terms of bandwidth and matching thickness, the reflection loss intensity cannot meet the strong absorption of electromagnetic waves by the absorber (Fig. 5d1, d2).

In Fig. 6c, e, the maximum *EAB* of Co_3_SnC_0.7_/CNF-700 is concentrated at the high matching thickness, while the EAB of Co_3_SnC_0.7_/CNF-800 and Co_3_SnC_0.7_/CNF-900 is inclined to thin thickness. The lowest reflection loss at different thickness can be observed in Fig. 6d, f. In the lower thickness range (1.7–2.7 mm), the minimum reflection loss of CoSnO_3_/PANF is greater than − 10 dB. Although the dielectric parameters of pure carbon nanofibers have been improved after carbonization, a small amount of interface polarization and defects determines the undesirable reflection loss of the material at low thickness. Compared with the above comparison samples, Co_3_SnC_0.7_/CNF-800 exhibits stronger electromagnetic attenuation characteristics, and the lowest reflection loss is less than − 20 dB in range of 2.0–2.7 mm. Especially at a thickness of 2.3 mm, the lowest *RL* of Co_3_SnC_0.7_/CNF reaches − 51.7 dB and the maximum *EAB* is 6.32 GHz. In addition, the *EAB* of the absorber reaches a peak value of 7.44 GHz at 2.5 mm.

**Fig. 7:**
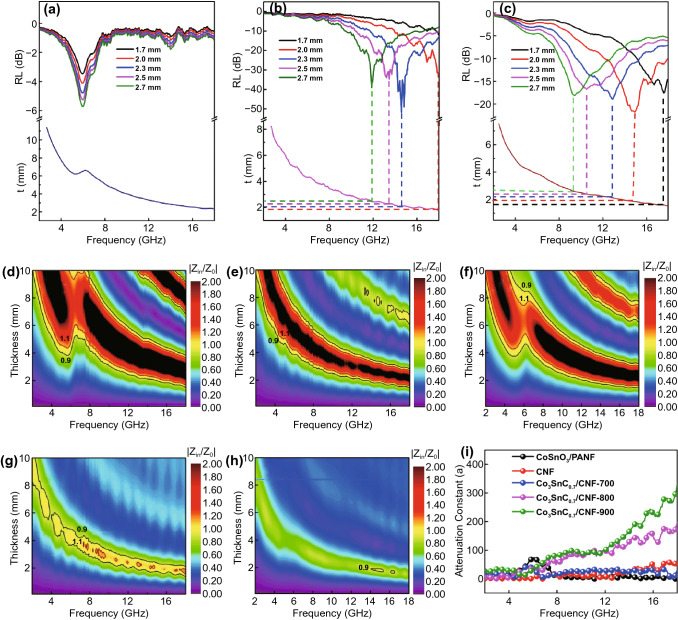
Dependence of matching thickness (*t*_m_) on matching frequency (*f*_*m*_) at wavelengths of *λ*/4 for Co_3_SnC_0.7_/CNF-700 (**a**), Co_3_SnC_0.7_/CNF-800 (**b**), Co_3_SnC_0.7_/CNF-900 (**c**); impedance matching |*Z*_in_/*Z*_0_| of CoSnO_3_/PANF (**d**), CNF (**e**), Co_3_SnC_0.7_/CNF-700 (**f**), Co_3_SnC_0.7_/CNF-800 (**g**), Co_3_SnC_0.7_/CNF-900 (**h**); the attenuation constant of five samples (**i**)

As the matching thickness increases, the frequency corresponding to the minimum reflection loss of Co_3_SnC_0.7_/CNF gradually shifts to the low frequency (Fig. 7a–c), which is concerned with the quarter-wavelength theory [49–51]:

3 $$t_{m} = n\lambda /4 = \frac{nc}{{4f_{m} \sqrt {\left| {\mu_{r} } \right|\left| {\varepsilon_{r} } \right|} }}\,\,\,\left( {n = 1,\,3,\,5 \ldots } \right)$$

Among them, *f*_*m*_ represents the frequency corresponding to the minimum reflection loss values and *t*_*m*_ is the matched thickness of the samples. It can be noted that the matching thickness corresponds to the calculated thickness. The attenuation EMW ability of absorber can be evaluated by the attenuation constant (*α*), which is derived from the following formula [52, 53]:

4 $$\alpha = \frac{\sqrt 2 \pi f}{c}\sqrt {\left( {\mu^{\prime \prime } \varepsilon^{\prime \prime } - \mu^{\prime } \varepsilon^{\prime } } \right) + \sqrt {\left( {\mu^{\prime \prime } \varepsilon^{\prime \prime } - \mu^{\prime } \varepsilon^{\prime } } \right)^{2} + \left( {\mu^{\prime } \varepsilon^{\prime \prime } - \mu^{\prime \prime } \varepsilon^{\prime } } \right)^{2} } }$$

According to Fig. 7i, CoSnO_3_/PANF has a lower attenuation constant at 2–18 GHz, and there are slight fluctuations in the frequency range. As the frequency of the sample CNF increases, the attenuation constant has an upward trend and eventually it reaches around 70 at high frequencies. Obvious fluctuations can be observed from the attenuation constant curve of the Co_3_SnC_0.7_/CNF composites. The value of *α* rises stably from 5 to 60 over the low frequency (2–8 GHz) and follows by a rapidly increase from 60 to 196 between 8 and 18 GHz. Thus, all different annealing temperatures of Co_3_SnC_0.7_ composites exhibit outstanding attenuation capability for dissipating EM energy.

In addition to the attenuation constant, the normalized characteristic impedance $\left( {Z = \left| {Z_{{{\text{in}}}} /Z_{0} } \right|} \right)$ of the absorber is also one of the important means to characterize the EMW absorption performance. The impedance relationship at different frequencies and thicknesses is vividly shown in Fig. 7. The impedance matching of the EMW absorber usually relies on the change of the *Z* value. When the *Z* value is close to 1, it indicates that there is almost no EMW reflection on the surface when the EMW enters the internal of absorbers. The 2D contour maps of the *Z* value for CoSnO_3_/PANF, CNF, and Co_3_SnC_0.7_/CNF composites are shown in Fig. 7d–h. According to the impedance distribution area, the yellow filled part indicates that the impedance matching value is closer to 1. The poor impedance matching of CoSnO_3_/PANF can be observed by a lot of black areas (*Z* > 2), while the yellow area is relatively narrow in Fig. 7d. As for CNF, 2D contour map (Fig. 7e) mainly composed of the green and black areas indicates that in most cases the value of *Z* is below 0.8 or higher than 2. Owing to the non-magnetic properties of the fibers, the electromagnetic wave absorption performance is strongly hindered by the unity of electromagnetic wave attenuation mechanism and the mismatch of electromagnetic wave input impedance. At the same time, the impedance matching of Co_3_SnC_0.7_/CNF-800 is evidently optimizing by the introduction transition metal (Fig. 7g). Especially around 2 mm, the value of impedance matching approaches 1 in the range of 12–18 GHz and more prominent electromagnetic wave absorption performance of the sample Co_3_SnC_0.7_/CNF-800 is confirmed.

**Fig. 8:**
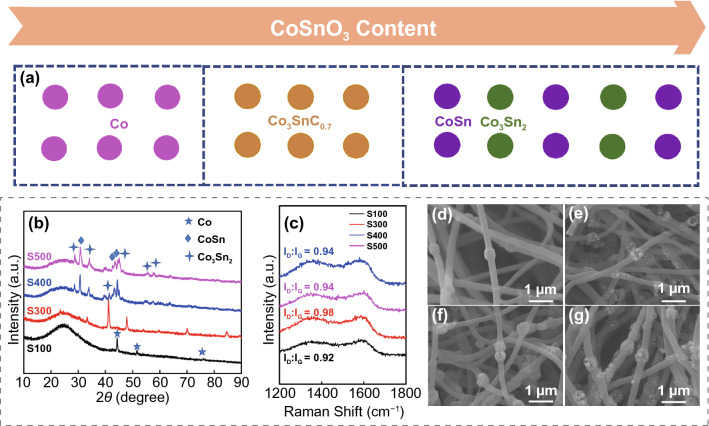
The relationship between CoSnO_3_ doping amount and final products phase transition (**a**); the XRD (**b**) and Raman (**c**) spectrums of S100, S300, S400, S500; the SEM images of S100 (**d**), S300 (**e**), S400 (**f**), S500 (**g**)

When the ratio of CoSnO_3_ and PAN changes, the crystalline state of the samples will transform during the carbonization process and it can be observed from XRD in Fig. 8b. After the annealing of CoSnO_3_/PANF with 100 mg CoSnO_3_ content, only obvious diffraction peaks of Co particles can be found in the pattern. With the increase of the content of CoSnO_3_ (400 and 500 mg), two metal alloy phases (CoSn and Co_3_Sn_2_) are gradually formed in the final product. It is related to the high polymer enrichment state around the metal nanoparticles during the annealing treatment. The Raman of samples S100, S300, S400, S500 are shown in Fig. 8c, and the ratio of *I*_D_–*I*_G_ is close to the sample of Co_3_SnC_0.7_/CNF-800, which confirms that the metal phase transition has less influence on the degree of graphitization of the sample. From Fig. S6, it can be found the distribution of nanoparticles in CoSnO_3_/PANF fibers changing with different amount of CoSnO_3_. However, excessive addition of CoSnO_3_ will lead to the agglomeration of the nanoparticles in fibers (Fig. S6b–f).

**Fig. 9:**
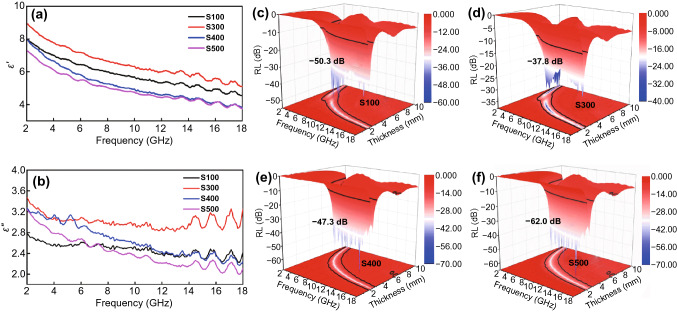
The curves of dielectric parameter (**a, b**) for S100, S300, S400, S500; the 3D reflection loss of S100 (**c**), S300 (**d**), S400 (**e**), S500 (**f**)

The dielectric real part of the samples S100–S500 (Fig. 9a) gradually increases and then decreases, which can be attributed to the change of the doping amount of CoSnO_3_. When the doping amount of CoSnO_3_ is low, it is difficult for the samples to form lots of interface polarization [54, 55]. Excessive content could lead to nanoparticle accumulation, which cannot effectively transport the space charge on the particle surface. It can be seen from Fig. 9c–f that the electromagnetic wave absorption performance of samples (S100, S300, S400, S500) is also excellent, especially in terms of reflection loss that the sample of S500 reaches − 62.0 dB, but the comprehensive performance such as effective bandwidth and matching thickness is lower than Co_3_SnC_0.7_/CNF-800.

### Microwave Absorption Mechanism

**Fig. 10:**
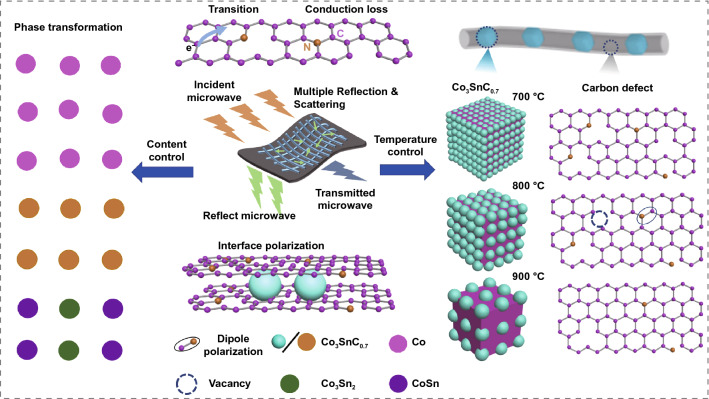
Schematic illustration revealing the mechanisms involved of the EMW wave absorption for the Co_3_SnC_0.7_/CNF composites

Based on the above results, the nanofiber composites exhibit remarkable wave absorption performance with thin thickness, strong absorption, and wide bandwidth. The possible EMW absorption mechanism for Co_3_SnC_0.7_/CNF composites is depicted in Fig. 10. Firstly, nanofibers can prevent the aggregation of CoSnO_3_ particles fillers, and the expanded inner diameter of nanofibers prolongs the reflection path of electromagnetic waves [56, 57]. After carbonization, the necklace structure formed by fibers and particles not only produces a large number of interface polarization effects, but also plays a role in blocking the propagation of electromagnetic waves. The amount of interface polarization can be qualitatively analyzed by adjusting the doping content of CoSnO_3_ in nanofibers. In Fig. S7, when the precursors with different CoSnO_3_ doping amounts are calcined at 800 °C, the performance change trend is small, which confirms that the interface polarization does not play a major role in the attenuation of electromagnetic waves. According to the dielectric real part curve of the sample in Fig. 4a, it is observed that the *ε*′ values of the samples Co_3_SnC_0.7_/CNF-700, Co_3_SnC_0.7_/CNF-800, and Co_3_SnC_0.7_/CNF-900 fluctuate slightly at high frequency. It confirms that partial polarization process occurs in the sample, which originates from the potential difference formed among the het8erointerfaces and the uneven charge distribution around the defect caused the generation of polarization center [58–60]. The excited electrons on the surface of Co_3_SnC_0.7_ solid particles or fibers migrate longitudinally or transition to the adjacent fiber surface through the conductive network, and the resulting conduction loss can attenuate electromagnetic waves by converting electromagnetic energy into thermal energy through the Joule effect [61–63]. As the temperature increases, the nanofibers are more inclined to form a conductive network, which is beneficial to the conduction loss. However, when the temperature is too high, the complex dielectric parameters of the sample and the complex magnetic permeability are unbalanced, resulting in mismatch of sample impedance. Since the inside of the absorber is unable to absorb the propagating electromagnetic waves effectively, a satisfactory absorption effect cannot be obtained. Therefore, the conduction loss of the composites is considered to play a major role in the entire dielectric loss process. On the other hand, Co_3_SnC_0.7_ can provide magnetic losses mainly originating from magnetic resonance such as natural resonance. In addition, combining metal oxides with fibers by electrospinning successfully improved the impedance matching of the composites. The distribution of metal particles inside the fibers will change with the feeding CoSnO_3_ content, thereby affecting the interfacial polarization. Due to the fixed calcination temperature, the electrical conductivity of the samples varied slightly. Therefore, there is a limited change in the absorption of nanofiber composites with conductivity loss as the main loss mechanism. Notably, a green metal-matrix fiber composite could be an excellent candidate for a high-performance electromagnetic wave-absorbing material.

## Conclusion

In summary, one-dimensional continuous Co_3_SnC_0.7_/CNF composites with tunable EMW absorption properties were prepared by electrospinning. The well-designed structure containing one-dimensional carbon nanofibers and hollow Co_3_SnC_0.7_ nanoparticles with a magnetic loss mechanism achieves high electromagnetic energy loss efficiency and good electromagnetic absorption impedance matching. In particular, the sample Co_3_SnC_0.7_/CNF-800 obtains strong dielectric loss through high conduction loss and moderate polarization loss, and cooperates with the magnetic loss of nanoparticles to improve the electromagnetic wave absorption performance of the composite. By comparison with recent related studies (Table S1), the Co_3_SnC_0.7_/CNF-800 composite exhibits a strong absorption capacity of − 51.2 dB at 14.56 GHz with a thickness of only 2.3 mm, which is superior to most reported nanofiber absorbers. The electromagnetic wave properties of one-dimensional nanocomposites are mainly determined by their dielectric properties, of which the conductivity loss is the dominant one. The combination of multi-component metal particles and carbon nanofibers provides a low-cost and sustainable strategy for the preparation of ultralight electromagnetic wave absorbers with excellent electromagnetic wave absorption properties. Therefore, the rational design of hollow CoSnO_3_ nanoparticles encapsulated in carbon nanofibers is pyrolyzed, and the product has stronger electromagnetic wave energy dissipation capacity and appropriate impedance matching with excellent electromagnetic absorption performance.

### Supplementary Information

Below is the link to the electronic supplementary material.

Supplementary file1 (PDF 795 KB)
