# Supplementary material for: Multicomponent Nanoparticles Synergistic One-Dimensional Nanofibers as Heterostructure Absorbers for Tunable and Efficient Microwave Absorption
Source: Nanomicro Lett. 2022 Dec 15;15:13. doi: 10.1007/s40820-022-00986-3 (PMC9755410; doi:10.1007/s40820-022-00986-3)
Supplement: Supplementary file 1 — Supplementary file1 (PDF 795 KB) [file 40820_2022_986_MOESM1_ESM.pdf]

Supporting Information for

Multicomponent Nanoparticles Synergistic One-Dimensional Nanofibers as Heterostructure Absorbers for Tunable and Efficient Microwave Absorption

Chenxi Wang[1, #],Yue Liu[1, #], Zirui Jia[2, 3, #, *], Wanru Zhao[1], Guanglei Wu[1, *]

[1]Institute of Materials for Energy and Environment, State Key Laboratory of Bio-fibers and Eco-textiles, College of Materials Science and Engineering, Qingdao University, Qingdao 266071, P. R. China

[2]College of Chemistry and Chemical Engineering, Qingdao University, Shandong, Qingdao 266071, P. R. China

[3]Weihai Innovation Institute, Qingdao University, Shandong, Qingdao 264200, P. R. China

[#]Chenxi Wang,Yue Liu, and Zirui Jia contributed equally to this work.

*Corresponding authors. E-mail: jiazirui@qdu.edu.cn (Z. Jia); wuguanglei@qdu.edu.cn or wuguanglei@mail.xjtu.edu.cn (G. Wu)

Supplementary Figures and Table

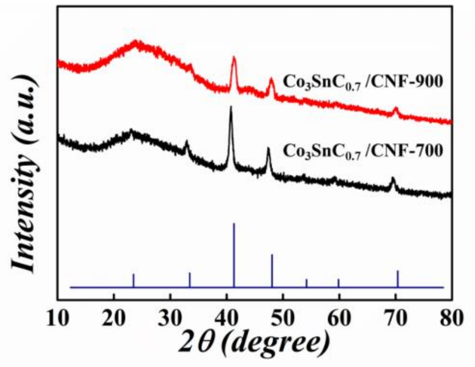

Fig. S1 XRD patterns of samples $Co_3SnC_{0.7}$/CNF-700 and $Co_3SnC_{0.7}$/CNF-900

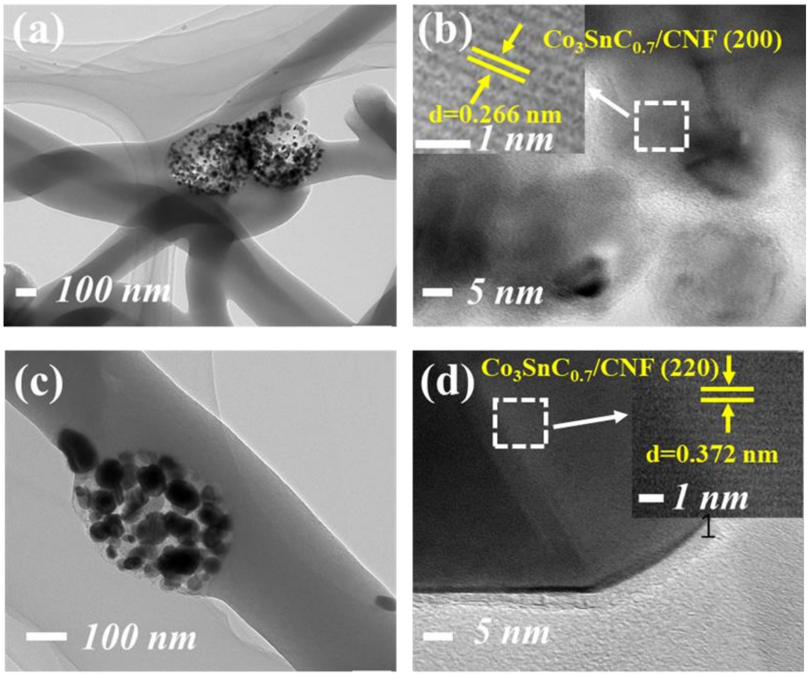

Fig. S2 The TEM images of $Co_3SnC_{0.7}$/CNF-700 (**a, b**) and $Co_3SnC_{0.7}$/CNF-900 (**c, d**)

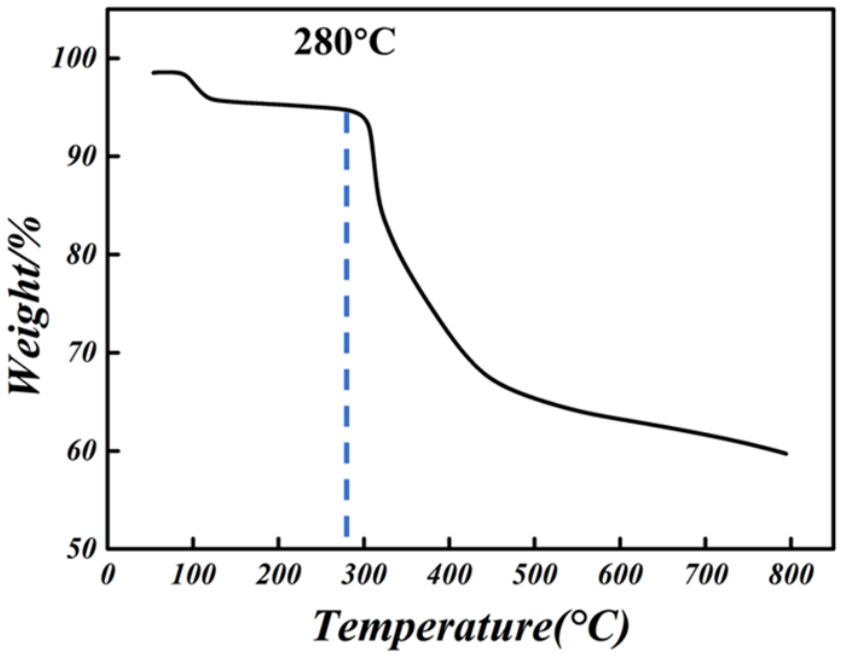

Fig. S3 Thermogravimetric curve of samples $CoSnO_3$/PANF

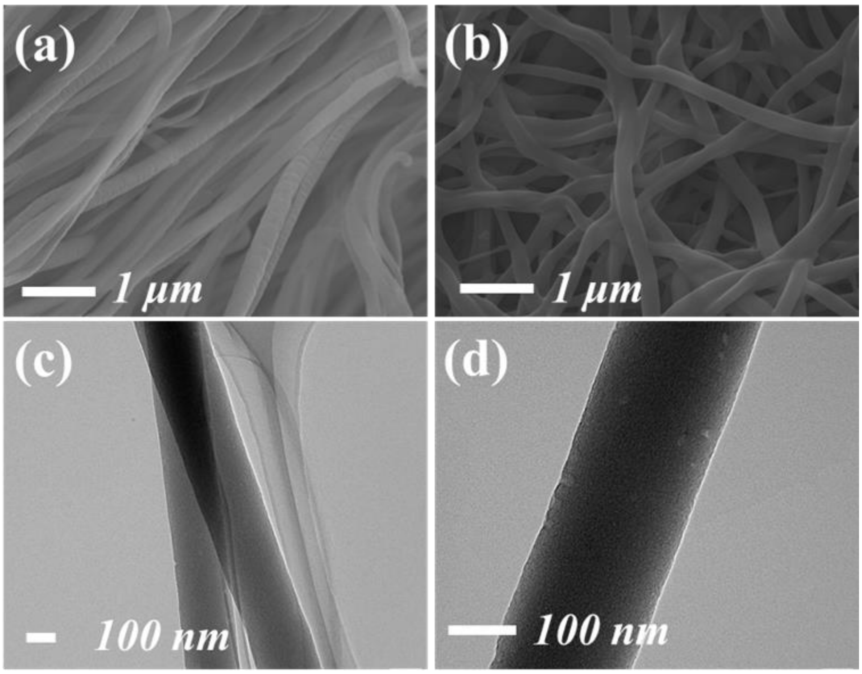

Fig. S4 SEM images of PANF (**a**), CNF (**b**); The TEM images of CNF (**c, d**)

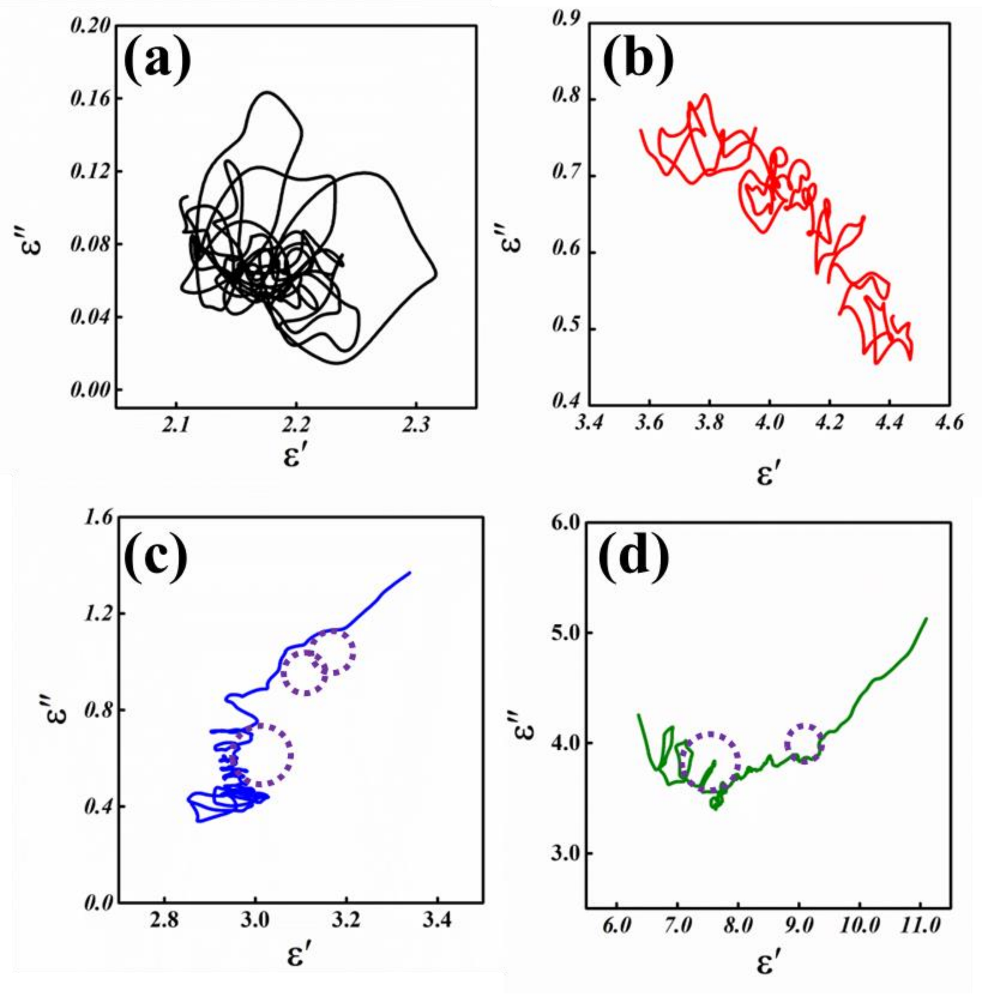

Fig. S5 The Cole-Cole curves of $CoSnO_3$/PANF (**a**), CNF (**b**), $Co_3SnC_{0.7}$/CNF-700 (**c**) and $Co_3SnC_{0.7}$/CNF-900 (**d**)

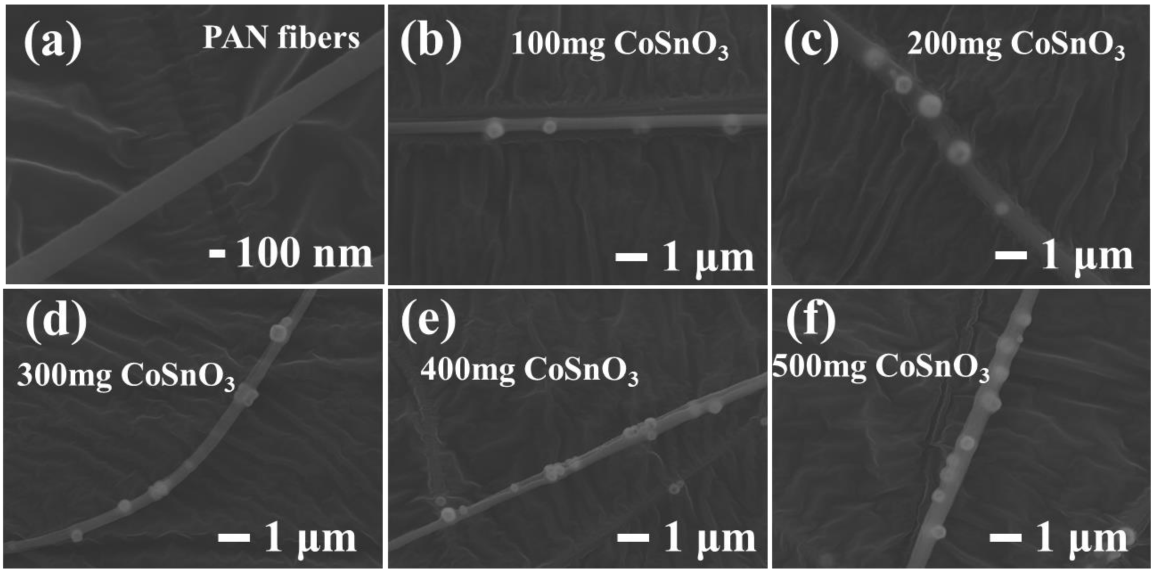

Fig. S6 The SEM images of $CoSnO_3$/PANF with 0 mg (**a**), 100 mg (**b**), 200 mg (**c**), 300 mg (**d**), 400 mg (**e**) and 500 mg (**f**) $CoSnO_3$ content

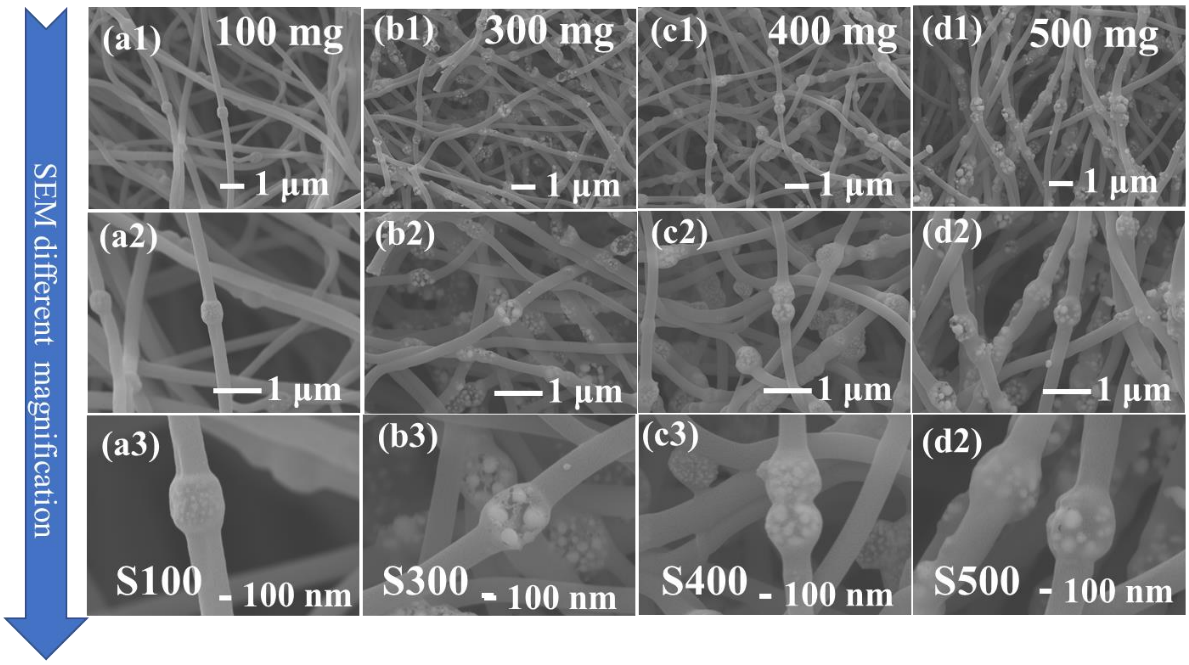

Fig. S7 The different magnifications SEM images of S100 (**a1-3**), S300 (**b1-3**), S400 mg (**c1-3**) and S500 (**d1-3**)

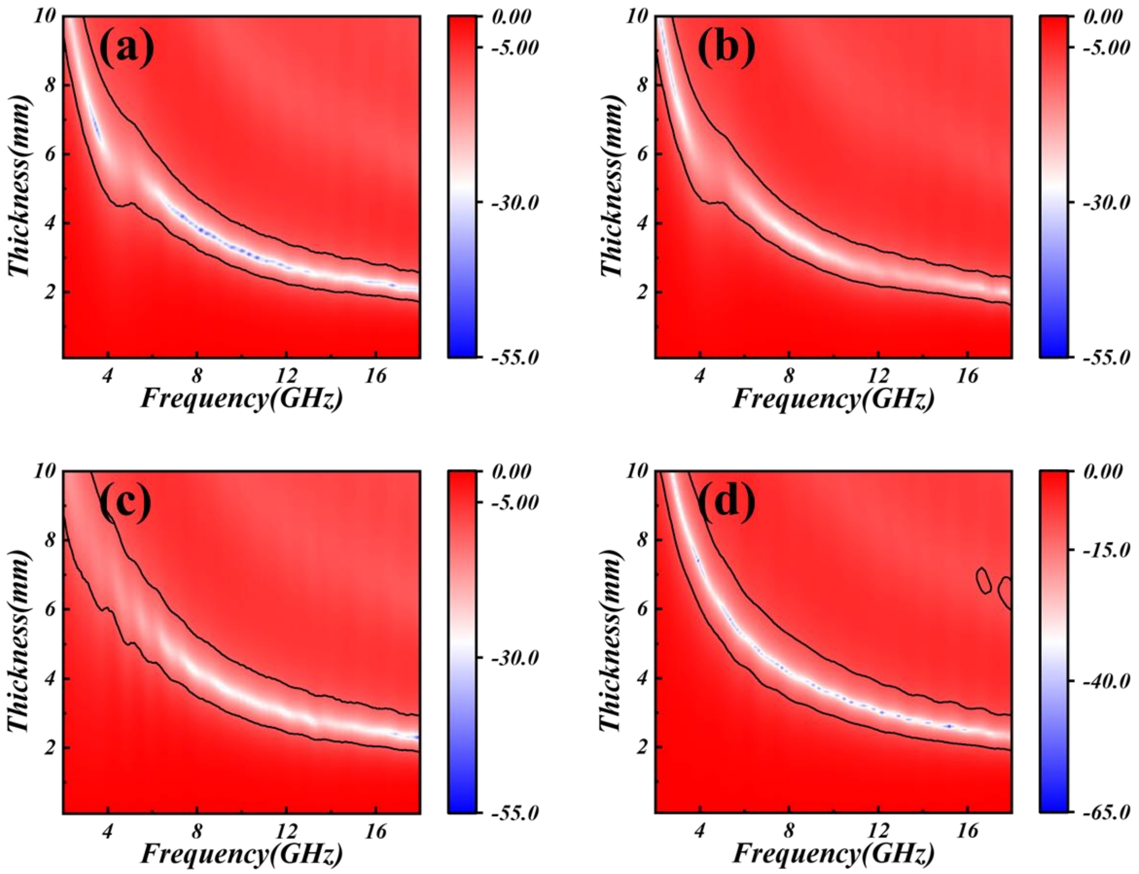

Fig. S8 2D effective bandwidth of S100 (**a**), S100 (**b**), S100 (**c**), S100 (**d**)

Table S1 Electromagnetic wave absorption performance of one-dimensional carbon matrix composites absorbers reported in previous studies and this work

**EMW absorbers**	**RL/dB**	**t_m(mm)**	**f_E/GHz**	**Refs.**
CF@1T/2H -MoS_2	-43	2.7	8.75 (3.5 mm)	[6]
CF@MXene@MoS_2	-61.51	3.5	7.6 (2.1 mm)	[16]
MMC	-51.6	1.6	4.6	[17]
CNT/SiC_f	-62.5	4	8.8 GHz	[23]
HCF@CZ-CNTs	-53.5	2.9	8.02 (2 mm)	[26]
Co-LDHs/SCFs	-40.4	2.0	6.5 (2.1 mm)	[27]
CN-ABF	-75.19	2.66	4.56 (2.66 mm)	[29]
Co@NCNTs/CF	-57.8	2	4.5 (2 mm)	[30]
SiC@C@PPy	-59.32	3.01	8.4 (2.78 mm)	[45]
ZrO_2-SiC/SiO_2	-18.1	10	5.52	[48]
CNTs/CF	-44.46	3	14.24 (0.5-6.0 mm)	[56]
$Co_3SnC_{0.7}$/CNF-700	-21.6	7.2	6.56 (8.0 mm)	This work
$Co_3SnC_{0.7}$/CNF-800	-51.7	2.3	7.44 (2.5 mm)	This work
$Co_3SnC_{0.7}$/CNF-900	-21.7	2.0	5.92 (2.2 mm)	This work
S400	-47.3	2.3	8.08 (2.6 mm)	This work
S500	-62.0	2.6	8.0 (2.9 mm)	This work
